# Opto-thermal technologies for microscopic analysis of cellular temperature-sensing systems

**DOI:** 10.1007/s12551-021-00854-1

**Published:** 2021-11-03

**Authors:** Kotaro Oyama, Shuya Ishii, Madoka Suzuki

**Affiliations:** 1 Quantum Beam Science Research Directorate, National Institutes for Quantum Science and Technology (QST), 1233 Watanukimachi, Takasaki, Gunma, 370-1292 Japan; 2 PRESTO, Japan Science and Technology Agency, 4-1-8 Honcho, Kawaguchi, Saitama, 332-0012 Japan; 3 Quantum Life and Medical Science Directorate, National Institutes for Quantum Science and Technology (QST), 4-9-1 Anagawa, Inage, Chiba, 263-8555 Japan; 4 Institute for Protein Research, Osaka University, 3-2 Yamadaoka, Suita, Osaka 565-0871 Japan

**Keywords:** Microscopy, Optical microheating, Temperature sensitivity, Temperature gradient, Thermometry

## Abstract

Could enzymatic activities and their cooperative functions act as cellular temperature-sensing systems? This review introduces recent opto-thermal technologies for microscopic analyses of various types of cellular temperature-sensing system. Optical microheating technologies have been developed for local and rapid temperature manipulations at the cellular level. Advanced luminescent thermometers visualize the dynamics of cellular local temperature in space and time during microheating. An optical heater and thermometer can be combined into one smart nanomaterial that demonstrates hybrid function. These technologies have revealed a variety of cellular responses to spatial and temporal changes in temperature. Spatial temperature gradients cause asymmetric deformations during mitosis and neurite outgrowth. Rapid changes in temperature causes imbalance of intracellular Ca^2+^ homeostasis and membrane potential. Among those responses, heat-induced muscle contractions are highlighted. It is also demonstrated that the short-term heating hyperactivates molecular motors to exceed their maximal activities at optimal temperatures. We discuss future prospects for opto-thermal manipulation of cellular functions and contributions to obtain a deeper understanding of the mechanisms of cellular temperature-sensing systems.

## Introduction

Sensing the temperature is an essential activity for life. Humans sense a variety of temperatures of air, water, and food in their daily lives, such as sauna (~ 100 °C), hot tea (~ 60 °C), comfortable shower (~ 40 °C), iced coffee (~ 4 °C), and ice cream (~ − 10 °C). We also sense our own internal temperature to maintain our body temperature. If the temperature-sensing system is dysfunctional, we cannot predict threats such as thermal injury and hypo- and hyperthermia.

It has been shown that living organisms are equipped with various different temperature sensors at the cellular level. The well-known temperature sensors are the thermo transient receptor potential (TRP) channels, which are the temperature-sensitive ion channels (Patapoutian et al. 2003). These channels are characterized by their high temperature sensitivities; the *Q*_10_ values of the TRP channels, which describe the rate of change in the current amplitude when the temperature is elevated by 10 °C, exceed 7 (Vriens et al. 2014). Furthermore, biochemical processes are temperature-sensitive in general (Elias et al. 2014). For example, ${Q}_{10}\approx 1-3$ for typical enzymatic reactions and ion channels, with the exception of thermo TRP channels. We also need to bear in mind that cellular systems involve coordinated functions of proteins. The Ca^2+^ channels, exchangers, pumps, and Ca^2+^-binding proteins maintain intracellular Ca^2+^ homeostasis and Ca^2+^ signaling (Berridge et al. 2003). The temperature sensitivity of the whole Ca^2+^ regulatory system may be a non-linear combination of the sensitivities of individual reactions, so it is usually difficult to predict until examined.

This review focuses on optical methods for manipulating the local temperature of cells to directly control a variety of cellular temperature-sensing systems. Optical heating is suitable for analyzing cellular temperature-sensing systems for the following reasons. First, it is well compatible with imaging-based analyses using optical microscopes. Second, it is free from the focus drift caused by the thermal expansion of materials such as plastics, glasses, and metallic components, which occurs when they are heated globally. We begin by introducing the optical microheaters and thermometers that have been used in microheating studies. Unique temperature-sensing systems in cells have been revealed and manipulated by these opto-thermal technologies, especially for muscle contractions, which is reviewed in this paper, followed by discussion on future prospects.

## Microscopic temperature manipulation

Pioneering studies have used a macro-heater or macro-cooler to produce spatial temperature gradients in microscopic areas. For instance, the macro-heater and macro-cooler comprise two copper fins connected with either hot or cold reservoirs (Ishizaka 1969). A linear temperature gradient of 1.5–6.5 °C per 100 μm was created over a grasshopper spermatocyte by adjusting the gap between the pair of fins. Nicklas fabricated a “microheater” with a resistance wire heater on a glass needle to apply a localized temperature gradient to a single cell (Nicklas 1973). The wire of a thickness of about 1.5–2 μm was bent in a U-shape of a diameter of 100 μm at the tip of the microheater. The temperature gradient was 10 °C and 15 °C in 10 μm and 50 μm from the microheater, respectively.

A temporal temperature gradient has been produced by optical heating. Optical temperature-jump (T-jump) methods are frequently used in studies of protein thermodynamics. For instance, a water-soluble triphenylmethane dye crystal violet was heated by a 532-nm laser pulse to induce the unfolding of RNase A (Phillips et al. 1995). The rate of temperature rise was 10 °C per 70 ps. Microscopic analyses with T-jump methods have enabled evaluation of the tension response of muscle fibers to fast temperature rises (Ranatunga 2018). Compared with the relatively fast temperature rise, the uniform heating of solutions with T-jump methods results in slow recovery to the initial temperature (~ 10 s) (Goldman et al. 1987).

**Fig. 1:**
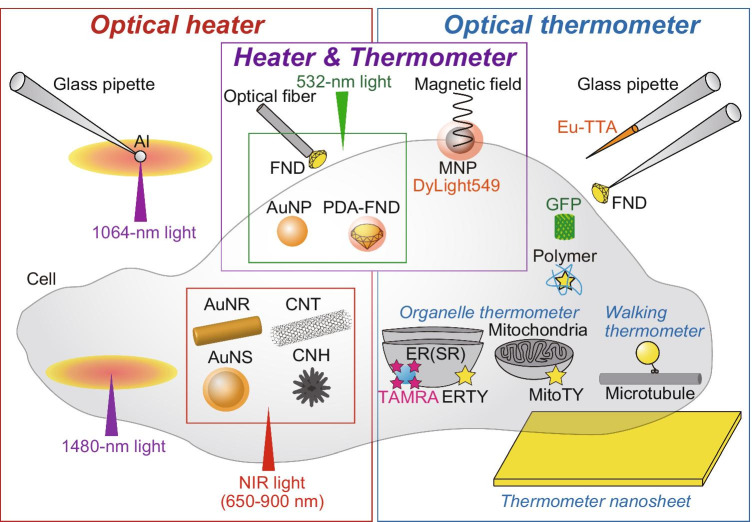
Optical microheaters and thermometers for cell analyses. *Left*, optical heating of aluminum (Al) particles attached to the tip of a glass micropipette generates square-shaped heat pulses (Zeeb et al. 2004). Water-absorbable light can heat the cells directly without materials. Gold nanorod (AuNR), gold nanoshell (AuNS), carbon nanotube (CNT), and carbon nanohorn (CNH) are excited by near-infrared (NIR) light (see text for details). *Right*, glass pipette that either encloses luminescent thermometer europium (III) thenoyltrifluoroacetonate trihydrate (Eu-TTA) (Zeeb et al. 2004) or attaches a fluorescent nanodiamond (FND) at the tip (Romshin et al. 2021) detect local temperature in extracellular solution. Thermometer nanosheet containing Eu-TTA visualizes surface temperature of cells (Itoh et al. 2014; Oyama et al. 2020). Temperature-sensitive fluorescent polymer (Tseeb et al. 2009) and green fluorescent protein (GFP) (Kamei et al. 2009) and its sophisticated derivatives (Nakano et al. 2017; Vu et al. 2021) are used as intracellular thermometers in microheating studies. Thermometer nanoparticles are enclosed in endosomes and transported along microtubules (named “walking thermometer”) (Oyama et al. 2012b). “Organelle thermometers” such as ER thermo yellow (ERTY) (Arai et al. 2014), Mito thermo yellow (MitoTY) (Arai et al. 2015b), and 5(6)-carboxytetramethylrhodamine (TAMRA)-azide (Hou et al. 2016) are targeted to specific organelles and can visualize the steep temperature gradient in cells during heating. *Center*, hybrid materials working as both heaters and thermometers have been developed with FNDs such as an FND attached to the tip of an optical fiber (Fedotov et al. 2015) or FNDs coated with the photothermal agent polydopamine (PDA) (Sotoma et al. 2021). Gold nanoparticles (AuNPs) are excited, and the changes of refractive index of the medium are probed for temperature measurement (Song et al. 2021). Magnetic nanoparticles (MNPs) covered with fluorescent thermometer dye DyLight594 are excited by radio-frequency magnetic field (Huang et al. 2010)

Optical microheaters have resolved this issue by decreasing the volume of the heat source (Fig. 1). An early attempt at this was “temperature pulse microscopy” using an aluminum aggregate (dimension ~ 10 μm) on a glass coverslip. The aggregate was heated by focusing a 1053-nm laser light to produce a concentric temperature gradient up to 2 °C μm^−1^ around the aggregate (Kato et al. 1999). The temperature gradient could be removed quickly by terminating the laser light irradiation. Square-wave heat pulses (originally referred to as “temperature pulses”) were created with rise and fall times of ~ 10 ms. If the aluminum aggregate was attached to the tip of a glass micropipette (tip ϕ ~ 1 μm) on micro manipulators, the heat source could be positioned at arbitrary locations (Zeeb et al. 2004).

Optical heating of nanomaterials is an effective strategy to minimize the volume of the heat source. Gold nanospheres illuminated by 532-nm laser light were shown to heat human embryonic fibroblast WS1 cells locally (Kucsko et al. 2013). Owing to the low absorption of near-infrared (NIR) light (~ 650–900 nm) by biological samples (Weissleder 2001), NIR-absorbable gold nanoshells (Loo et al. 2005; Gobin et al. 2007; Marino et al. 2017), gold nanonods (Huang et al. 2006; Eom et al. 2014; Yoo et al. 2014; Yong et al. 2014), and star-shaped gold nanoparticles (Zhu et al. 2021) have been developed for photothermal stimulation in deep tissues. NIR light was also used to heat carbon nanomaterials such as carbon nanotubes (Kam et al. 2005; Miyako et al. 2012b) and carbon nanohorns (Miyako et al. 2012a). Miyako et al. conjugated the NIR fluorescent dye IRDye800CW to carbon nanohorns to enhance the heat power of the nanomaterials in cells (Miyako et al. 2014).

Optical microheating without materials is convenient to target arbitrary positions, especially in vivo. Optical laser traps (optical tweezers) with 1064-nm laser light were reported to directly heat membranes of CHO cells with heating efficiency of ~ 1.15 °C per 100 mW (Liu et al. 1995). Water-absorbed laser light achieves heating more efficiently. Kamei et al. used 1480-nm laser light for heating acrylamide gel as an in vitro tissue model with a heating rate of ~ 1 °C mW^−1^ and then applied the method in living *C. elegans* to activate heat shock promoter, followed by the induction of gene expression only in targeted cells (Kamei et al. 2009).

## Microscopic temperature measurement

Thermocouples are commonly used to measure the temperature in solution, but they are too large for the scale of single cells. Therefore, in early studies, the temperature was visualized by the dewing point (31 °C) and freezing front (0 °C) (Ishizaka 1969) or estimated from the birefringence of mitotic spindles (Nicklas 1973).

The development of microscopic luminescence thermometry has constituted progress in fluorescence microscopy (Fig. 1). Temperature changes alter the luminescence properties of temperature probes such as intensity, absorption and emission spectra, polarization, and lifetime (Jaque and Vetrone 2012; Brites et al. 2012; Zhou et al. 2020). Microscopic temperature imaging in solution can be performed by relatively simple methods that detect the thermal quenching of water-soluble luminescent dyes such as rhodamine B (Ross et al. 2001), BCECF (Braun and Libchaber 2002), and tetramethylrhodamine or Alexa Fluor 555 conjugated to dextran (Oyama et al. 2015a). Luminescent nanosheets containing the temperature-sensitive dye europium (III) thenoyltrifluoroacetonate trihydrate (Eu-TTA) visualize the temperature distribution on the glass surface during optical microheating of solution (Itoh et al. 2014; Oyama et al. 2020). Romshin et al. determined the temperature gradient using a fluorescent nanodiamond (FND) attached to the tip of a glass micropipette (Romshin et al. 2021).

Luminescence properties of the temperature probes can also be affected by non-thermal environmental parameters such as pH. This issue can be resolved by a probe that has perfect robustness. For example, temperature-sensitive dye can be covered by another material that functions as a coating to protect the dye from environmental changes. We demonstrated this strategy experimentally for the first time, where Eu-TTA dye was enclosed in a glass micropipette (Zeeb et al. 2004). We further expanded this strategy by developing robust polymer-nanoparticles embedding temperature-sensitive luminescent dyes (Oyama et al. 2012b; Takei et al. 2014; Arai et al. 2015a; Ferdinandus et al. 2016).

Intracellular luminescent thermometry has been developed to detect cellular thermogenesis (Suzuki et al. 2016; Okabe et al. 2018; Zhou et al. 2020). The same luminescent thermometers are applicable in combination with microheating methodologies. Temperature-sensitive fluorescent polymers (Uchiyama et al. 2004) injected into HeLa cells demonstrated that the temperature in the cytoplasm rose and dropped within 300 and 100 ms, respectively, in response to a heat pulse, where the heat pulse was created by an aggregate of aluminum nanoparticles illuminated by a focused 1064-nm laser light (Tseeb et al. 2009). The 3D temperature distributions in acrylamide gel during heating with a 1480-nm laser light were visualized using *E. coli* overexpressing GFP (Kamei et al. 2009). Genetically encoded fluorescent thermometers measured the temperature changes in cytoplasm (Nakano et al. 2017) and nuclei (Vu et al. 2021) during opto-thermal microheating. FNDs were also demonstrated to detect temperature gradients in cells when intracellular gold nanoparticles were illuminated by 532-nm light (Kucsko et al. 2013) or when heated by focused 1480-nm laser light (Choi et al. 2020). Other examples are small molecules targeted to the endoplasmic reticulum (ER) (Arai et al. 2014) and mitochondria (Arai et al. 2015b), or fluorescent nanosensors targeted to ER by an intracellular bottom-up approach (Hou et al. 2016). These organelle-targeted fluorescent probes visualized the temperature gradients in respective organelles during local heating.

Two technologies of temperature manipulation and thermometry need to be combined in single hybrid nanomaterials to determine the temperature of the nanomaterial as a small heat source (Fig. 1). Superparamagnetic nanoparticles were coated with the temperature-sensitive dye DyLight549 to measure the temperature of the nanoparticle surface (Huang et al. 2010). Fluorescent diamonds have been used as hybrid materials in several ways. For instance, diamond microcrystal attached to the tip of an optical fiber was heated by a 532-nm laser (Fedotov et al. 2015). Nanohybrids of gold nanorod-FND (Tsai et al. 2015) were used to determine the rupture temperature of cell membranes (Tsai et al. 2017). Sotoma et al. coated FND with polydopamine demonstrating photothermal conversion. They successfully measured the intracellular thermal conductivity for the first time as about one-sixth of that of water, where significant variation of the value was also recognized (Sotoma et al. 2021). To map the possible inhomogeneity of the heat transfer in a cell, Au nanoparticles were pumped with a 532-nm laser for heating locally, while the localized temperature changes were probed using white light by detecting the temperature-dependent changes of the refractive index of the surrounding medium (Song et al. 2021).

## Cellular responses to spatial temperature gradient

The spatial gradient of the temperature is formed in tissues in the range of ~ 0.01–1 °C mm^−1^. For instance, the temperature gradients across skin and eye are 0.2–0.5 °C mm^−1^ (Bazett and McGlone 1927) and 0.1–1.3 °C mm^−1^ (Schwartz and Feller 1962), respectively. In rabbit oviduct, the temperature difference between the sperm storage site and fertilization site (the distance between two sites is ~ 100 mm) is increased from 0.8 to 1.6 °C after ovulation (Bahat et al. 2005). From these values, the temperature difference across a single cell (~ 10 μm) is calculated as ~ 0.0001–0.01 °C when a one-dimensional homogeneous temperature gradient is assumed. These tiny temperature gradients are known to affect cellular behaviors. Human sperm cells show thermotaxis; they respond to the temperature gradient of < 0.014 °C mm^−1^ and migrate toward the warmer side (Bahat et al. 2003, 2012). Surprisingly, the temperature difference in the cell body (46 μm) is < 0.0006 °C.

**Fig. 2:**
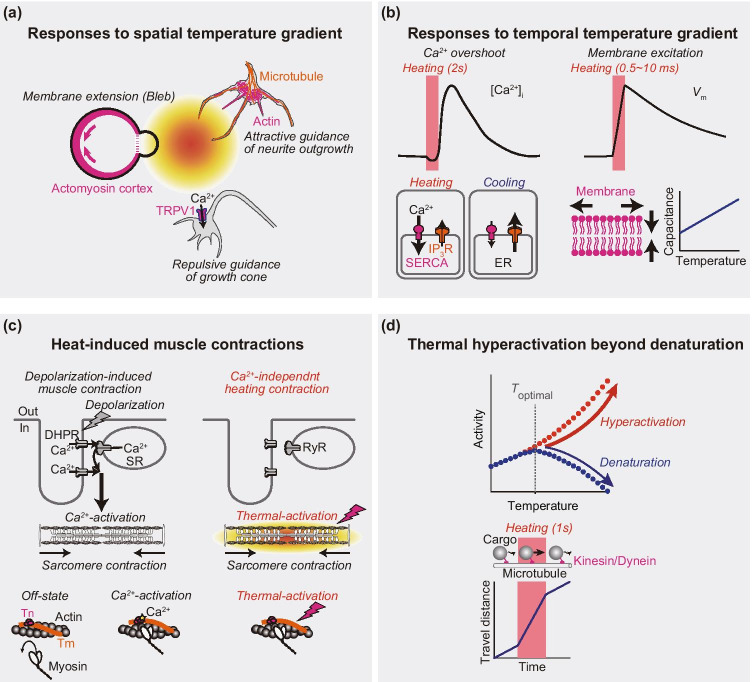
Cellular responses to spatial and temporal temperature gradients. **a** Responses to spatial temperature gradient. *Left*, spherical mitotic cells extend the plasma membrane (bleb) toward the heat source with asymmetric movement of actomyosin cortex (Oyama et al. 2015a). *Right*, neurites elongated toward the heat source with enhanced polymerization/sliding of microtubules and actin filaments (Oyama et al. 2015b). *Bottom*, repulsive guidance of growth cone is triggered by heat-activated Ca^2+^ influx through transient receptor potential channels (TRPV1) (Black et al. 2016). **b** Responses to temporal temperature gradient. *Left*, heat pulse elevates the intracellular concentration of Ca^2+^ ([Ca^2+^]_i_) due to Ca^2+^ release from intracellular Ca^2+^ store endoplasmic reticulum (ER) (Tseeb et al. 2009; Itoh et al. 2014). During heating, Ca^2+^ uptake by sarco-/endoplasmic reticulum Ca^2+^ ATPase (SERCA) is activated (larger arrow). At the end of heating, Ca^2+^ release through inositol trisphosphate receptors (IP_3_R) is enhanced (larger arrow). *Right*, rapid heating elevates membrane potential (*V*_m_) with capacitance increase (Shapiro et al. 2012; Liu et al. 2014) due to geometric changes of bilayer (arrows) (Plaksin et al. 2018). **c** Mechanisms of muscle contractions triggered by depolarization of sarcolemma (*left*) or Ca^2+^-independent thermal activation of contractile systems (*right*) (see text for details). **d** Thermal hyperactivation of molecular motors. Long exposure of temperature higher than optimal temperature (*T*_optimal_) decreases the enzymatic activity due to thermal denaturation (blue plots). Short heating enables an increase in enzymatic activity, which is higher than the maximal activity at *T*_optimal_ (red plots). This hyperactivation of molecular motors interacting with the cytoskeleton has been observed in vitro (Kato et al. 1999; Kawaguchi and Ishiwata 2001) and in cells (Oyama et al. 2012b).

A temperature gradient affects the process of cell division. A temperature gradient (6 °C per 100 μm) along the spindle of anaphase spermatocytes accelerates the development of the aster at the warmer side, moves the spindle toward the cooler side, and induces asymmetric division (Ishizaka 1969). Nicklas applied a steeper gradient than that of the previous study (15 °C per 50 μm) and observed accelerated chromosome separation at warmer side (Nicklas 1979). The plasma membrane at the hotter side of a mitotic HeLa cell is extended toward the heat source, termed a polar bleb, due to the asymmetric movement of actomyosin cortex by imbalanced actomyosin contractile forces (Oyama et al. 2015a) (Fig. 2a). The minimum temperature difference within the cell (~ 20 μm) to form the polar bleb was found to be 1.3 °C, or over 65 °C mm^−1^. Such a large gradient has not been observed in tissues, but it may be formed in thermal therapies using nano-/micromaterial heaters (Rajan and Sahu 2020; Liao et al. 2021).

Cellular response to the temperature gradient is also the subject of studies examining the mechanism in the optical guidance of neurite outgrowth. Ebbesen and Bruus calculated the temperature gradient during the optical guidance using an infrared (IR) laser light and proposed that the heat activation of TRP channels mediates the neuronal guidance (Ebbesen and Bruus 2012). Their conclusion matched the experimental findings; TPRV1 antagonist suppressed repulsive response of growth cone to the focused 785-nm laser light (Black et al. 2016). We observed the attraction of neurite outgrowth of rat hippocampal neurons toward the heat source that was formed by 1455-nm IR laser light (Oyama et al. 2015b). While the neurite outgrowth was independent of the TRP channels’ activity, substantial but non-essential contribution of Ca^2+^ influx was demonstrated. We proposed the mechanism based on heat-accelerated polymerization of actin filaments and microtubules and their sliding by molecular motors.

## Cellular responses to temporal temperature gradient

The previous section summarized cellular responses to the spatial temperature gradient that is formed in a steady state. Here, we show that the sudden changes in temperature can also cause various cellular responses that range from Ca^2+^ signaling to membrane excitation.

Rapid cooling induces intracellular [Ca^2+^] ([Ca^2+^]_i_) increases in various types of cell. For instance, rapid cooling from 36.5 °C to a temperature below 18 °C induces guinea pig cardiac muscle contractures, named *rapid cooling contractures* (RCC) (Kurihara and Sakai 1985). The maximum tension during RCC depends on the rate of cooling (Sakai and Kurihara 1974). The rapid cooling induces Ca^2+^ release from intracellular Ca^2+^ store sarcoplasmic reticulum (SR) mainly through ryanodine receptors (RyRs) (Protasi et al. 2004) and probably also through inositol trisphosphate receptors (IP_3_Rs) (Talon et al. 2000), both of which are SR Ca^2+^ release channels. Cooling elevates the open probability of RyRs (Sitsapesan et al. 1991). Additionally, cooling decreases the activity of sarco-/endoplasmic reticulum Ca^2+^ ATPase (SERCA) (Dode et al. 2001; Landeira-Fernandez et al. 2004), which is suggested to increase the net Ca^2+^ leak from SR. Rapid cooling is also known to induce [Ca^2+^]_i_ increases in *Paramecium* (Inoue and Nakaoka 1990) and in plant cells (Plieth et al. 1999; Nagel-Volkmann et al. 2009).

Interestingly, similar [Ca^2+^]_i_ increases are also induced by a heat pulse for several seconds. When a heat pulse for 2 s was applied to HeLa cells, there was a [Ca^2+^]_i_ decrease during heating, followed by its overshoot after the end of the heating (Tseeb et al. 2009) (Fig. 2b, upper left). The minimum temperature rise that was required to induce the Ca^2+^ overshoot was 1.5 °C at 22 °C, which was reduced to 0.2 °C at 37 °C. Human fibroblast WI-38 cells responded in a similar manner (Itoh et al. 2014). In both cells, the increase in [Ca^2+^]_i_ was suppressed by the inhibitors of IP_3_R, strongly suggesting that IP_3_R is the major Ca^2+^ release channel in the heat pulse-induced [Ca^2+^]_i_ increase. A plausible mechanism explaining this is as follows (Fig. 2b, lower left): (i) Heating elevates the net Ca^2+^ flow from the cytoplasm toward ER due to increased SERCA activity and probably decreased open probability of IP_3_R (similar temperature sensitivity of the open probability as proposed in RCC). The electrochemical potential of Ca^2+^ across the ER membrane is enhanced. (ii) At the end of heating, the net Ca^2+^ flow is quickly reversed as the activities of SERCA and IP_3_R immediately return to the pre-heating states. (iii) The Ca^2+^ leak that follows the enhanced Ca^2+^ gradient serves as the initial Ca^2+^ for a large Ca^2+^ leak known as the Ca^2+^-induced Ca^2+^ release of IP_3_R. In summary, the Ca^2+^ response observed here is a result of the asymmetry between the temperature sensitivities of Ca^2+^ pumps and Ca^2+^ release channels and their thermal perturbation.

Rapid heating also induces excitation of the cell membrane. Wells et al. previously observed IR stimulation of neuronal cells (Wells et al. 2005). They examined photochemical, photomechanical, and photothermal effects on sciatic nerve and concluded that the photothermal effect causes activation of the plasma membrane (Wells et al. 2007). Shapiro et al. showed that the electrical capacitance of the membrane without ion channels is increased by rapid heating (Shapiro et al. 2012) (Fig. 2b, right). They proposed a theoretical explanation for this based on the Gouy-Chapman-Stern theory (Genet et al. 2000) that rapid heating induces asymmetric charge displacements at the intracellular and extracellular sides of the plasma membrane. The rate of temperature rise was the key parameter for exciting the cell membrane of *C. elegans* (Liu et al. 2014), which was consistent with the model (Shapiro et al. 2012). Plaksin et al. pointed out an error in the theoretical modeling and proposed an alternative theory considering dimensional changes in the membrane (Plaksin et al. 2017, 2018) (Fig. 2b, right).

## Muscle contractions induced by heat pulses

IR cardiac stimulation is effective for the optical pacing of hearts. Smith et al. succeeded in inducing [Ca^2+^]_i_ transients in rat neonatal cardiomyocytes using a 780-nm femtosecond pulse laser light (Smith et al. 2008). Their efforts were based on their own findings that pulse laser light induced [Ca^2+^]_i_ increase in HeLa (Smith et al. 2001; Iwanaga et al. 2006) and PC12 cell lines (Smith et al. 2006). Pulsed 1875-nm laser light was also reported to achieve optical pacing of quail embryonic heart (Jenkins et al. 2010). The membrane excitation induced by opto-thermal stimulations (Shapiro et al. 2012) could trigger Ca^2+^ influx through sarcolemma voltage-sensitive Ca^2+^ channels and Ca^2+^ release from intracellular Ca^2+^ store SR, as well as physiological cardiac contractions (Bers 2002). Other Ca^2+^ sources are additionally suggested. Dittami et al. investigated the mechanism of [Ca^2+^]_i_ transients in rat neonatal cardiomyocytes evoked by pulsed IR light (1862 nm) and concluded that the major intracellular Ca^2+^ sources were mitochondria (Dittami et al. 2011). Similarly, inhibitors of mitochondrial Ca^2+^ cycling suppressed the [Ca^2+^]_i_ transients evoked by IR laser light in rat ganglion neurons (Lumbreras et al. 2014). Thus, mitochondria might also be the Ca^2+^ source of the [Ca^2+^]_i_ transients induced by opto-thermal stimulations in these cells.

Train of sub-second heat pulses over ~ 40 °C can induce repeated Ca^2+^-independent muscle contractions. Cardiomyocytes isolated from adult rats were also found to repeat the cycle of contraction and relaxation as a response to heat pulses (0.2 s) applied at 2.5 Hz (Oyama et al. 2012a). The contraction induced by heating from 36 to 41 °C was suppressed by a myosin II inhibitor, blebbistatin. The [Ca^2+^]_i_ was stable during the contraction. These results strongly suggest that the heating triggers the interaction of actin and myosin in a Ca^2+^-independent manner (Fig. 2c). Shintani et al. further investigated the effect of heating on sarcomere dynamics with high-precision measurement of sarcomere lengths in rat neonatal cardiomyocytes and found that heating to ~ 38 °C induced Ca^2+^-independent high-frequency (5–10 Hz) sarcomeric oscillations termed *hyperthermal sarcomeric oscillations* (HSOs) (Shintani et al. 2015).

What is the mechanism of these heat-induced Ca^2+^-independent muscle contractions? Physiological cardiac contractions are regulated by [Ca^2+^]_i_ as follows (Bers 2002) (Fig. 2c). (i) Depolarization of excited sarcolemma triggers Ca^2+^ influx through voltage-sensitive Ca^2+^ channel [dihydropyridine receptor (DHPR)] at the sarcolemma. (ii) The Ca^2+^ influx from extracellular space triggers intracellular Ca^2+^ release from SR. (iii) The Ca^2+^ influx and release increase [Ca^2+^]_i_, which promotes Ca^2+^ binding to troponin C (TnC) on thin filaments. (iv) The Ca^2+^ binding to TnC shifts the state of thin filaments to “on,” which allows the interaction of actomyosin. (v) Sarcomere shortening is initiated. (vi) As [Ca^2+^]_i_ decreases mainly due to Ca^2+^ uptake into SR by SERCA and Ca^2+^ efflux by sarcolemmal Na^+^/Ca^2+^ exchanger, Ca^2+^ dissociates from TnC and the state of thin filaments shifts to “off,” which blocks actomyosin interaction.

Heat-induced contraction without [Ca^2+^]_i_ increase could be explained from the perspective of Ca^2+^-independent thermal activation of thin filaments (Ishii et al. 2020). In an in vitro motility assay, reconstituted cardiac thin filaments slid on myosin in Ca^2+^-free solution when the temperature was increased over ~ 43 °C (Brunet et al. 2012). Optical rapid heating initiated the Ca^2+^-independent sliding within 30 ms (Ishii et al. 2019). These results show that actomyosin interaction is enhanced by heating without Ca^2+^. Interestingly, the sliding speed in Ca^2+^-free solution at 37 °C was about 30% of that in a Ca^2+^-activated state, suggesting that cardiac muscles are partially activated in the relaxed condition (diastole) at physiological temperature for rapid and efficient contraction in systole.

Similar Ca^2+^-independent contraction was also demonstrated using NIR laser light and the photothermal property of gold nanoshells that were internalized in myotubes differentiated from the skeletal muscle model C2C12 cell line (Marino et al. 2017). At least three processes could be suggested to explain the heat-activated muscle thin filaments. First, the regulatory proteins may have partially dissociated during heating. The complex of tropomyosin (Tm)-Tn has been shown to dissociate from actin filaments at temperatures above ~ 41 °C (Ishiwata 1978). Heating unfolds the coiled-coil domains of Tm and decreases the affinity with actin (Kremneva et al. 2003). Initiation of these processes on thin filaments could contribute to the Ca^2+^-independent activation of thin filaments during the heat pulses. Second, heating increases the affinity of TnC to Ca^2+^ (Gillis et al. 2000; Veltri et al. 2017), allowing for actomyosin interaction during heating at relatively low [Ca^2+^]_i_. Lastly, heating also elevates the affinity of actin and myosin (Highsmith 1977, 1978) and increases the number of force-generating crossbridges (Zhao and Kawai 1994); that is, crossbridge force generation is endothermic (Ranatunga 2018). These properties of actomyosin could result in the cooperative formation of crossbridges as well as the strong binding of myosin, which ensures the proximity of the myosin binding sites on the thin filament in the absence of Ca^2+^ (Lehman 2017; Geeves et al. 2019).

## Thermal hyperactivation of enzymes without denaturation

Enzymatic reactions of proteins are elevated by a temperature rise, as described by the Arrhenius equation, but proteins are inactivated due to thermal denaturation when overheated above the inherent optimal temperature (Daniel and Danson 2013) (Fig. 2d). However, protein denaturation is a time-dependent process. Enzymes exposed to temperatures above the optimal temperature could be hyperactivated at the beginning of the heating according to the Arrhenius equation, and they are then denatured if the heating continues thereafter. This scenario has been directly demonstrated in a microscopic experiment in vitro, where actomyosin motors were reversibly hyperactivated above physiological temperature (> 60 °C) for a short period (62.5 ms) of heating (Kato et al. 1999). The gliding velocity of microtubules over kinesin molecules in vitro was increased at the temperature up to 50 °C for 2 s by following the Arrhenius equation, whereas kinesins were inactivated after heating at 35 °C for 1 min (Kawaguchi and Ishiwata 2001). Heating to 100 °C for tens of nanoseconds (~ 40 ns) caused no apparent thermal denaturation of catalase, and the rate of inactivation at up to ~ 174 °C is consistent with the Arrhenius equation (Steel et al. 2006). In HeLa cells, the velocity of endosomes transported on microtubules by the molecular motor kinesin or dynein was increased during heating up to 47 °C for 1 s (Oyama et al. 2012b). Thus, a short period of heating (~ 2 s) hyperactivates enzymes to exceed their steady state maximal speed at the optimal temperature.

Thermal hyperactivation of enzymatic reactions may enable us to control cellular functions in an analog way by adjusting the amplitude of opto-thermal stimulation. This is in contrast with other optical manipulations such as optogenetics, which are usually based on on–off digital regulation. The method may also be applied to achieve enhanced performance of cells beyond the physiological level.

### Advantages and limitations of opto-thermal cellular manipulation

The combination of optical heating and cells engineered with the heat shock promoter-mediated gene expression systems is used for spatial and remote regulation of cellular activities (Kamei et al. 2009; Miyako et al. 2012a; Miller et al. 2018). Temperature-sensitive mutant of myosin II was also employed for local inactivation of the mutant in *C. elegans* embryos, and the division failures were induced in targeted cells (Hirsch et al. 2018). Moreover, opto-thermal cellular manipulation can target endogenous temperature-sensing systems (Table 1). It is not necessary to express light-sensitive proteins or introduce light-sensitive materials. Therefore, opto-thermal methods are suitable especially in in vivo applications and in non-model species and are even applicable for thermal therapy.

**Table 1:** Cellular responses to opto-thermal stimulations

Cellular response	Target	Optical heater (wavelength)	Types of temperature gradient (heating period)	Thermometer	Cellular thermosensor	Reference
Membrane extension	HeLa	CW laser (1455 nm)	Spatial (20 s)	Fluorescent dextran	Actomyosin cortex	Oyama et al. 2015a
Neurite outgrowth	Rat hippocampal neuron	CW laser (1455 nm)	Spatial (60 s)	Thermometer nanosheet	Cytoskeleton and molecular motors	Oyama et al. 2015b
Growth cone repulsive response	Rat cortical neuron	CW laser (750–1000 nm)	Spatial (> min)	IR camera	TRPV1	Black et al. 2016
[Ca^2+^]_i_ increase	HeLa	80-fs pulse laser (780 nm)	Temporal (125–500 ms)	-	Internal Ca^2+^ store	Smith et al. 2001*
[Ca^2+^]_i_ increase	HeLa	80-fs pulse laser (780 nm)	Temporal (13 ms)	-	ER	Iwanaga et al. 2006**
[Ca^2+^]_i_ increase	PC12	80-fs pulse laser (775 nm)	Temporal (13 ms)	-	-	Smith et al. 2006 *
[Ca^2+^]_i_ increase	Rat ganglion neuron	4-ms pulse laser (1863 nm)	Temporal (4 ms)	-	Mitochondria	Lumbreras et al. 2014**
[Ca^2+^]_i_ increase	HeLa	Al (1064 nm)	Temporal (2 s)	Eu-TTA in a glass pipette	ER (SERCA and IP_3_R)	Tseeb et al. 2009
[Ca^2+^]_i_ increase	WI-38	CW laser (1455 nm)	Temporal (2 s)	Thermometer nanosheet	ER (SERCA and IP_3_R)	Itoh et al. 2014
[Ca^2+^]_i_ increase	MCF-7, HeLa	Star-shaped AuNP (830 nm)	Temporal (39 ms)	-	Lysosome	Zhu et al. 2021**
Nerve excitation	Sciatic nerve (frog, rat)	Pulse laser (0.75–2.12 μm)	Temporal (5–5000 μs)	IR camera	Plasma membrane	Wells et al. 2007
Membrane excitation	Frog oocyte, HEK293T	Pulse laser (1869–1889 nm)	Temporal (0.1–10 ms)	Impedance of a glass pipette	Plasma membrane	Shapiro et al. 2012
Membrane excitation	*C. elegans*	Pulse laser (1862 nm)	Temporal (300–1500 μs)	Impedance of a glass pipette	Plasma membrane	Liu et al. 2014
Cardiac [Ca^2+^]_i_ increase and contraction	Rat neonatal cardiomyocytes	80-fs pulse laser (780 nm)	Temporal (8 ms)	-	-	Smith et al. 2008*
Cardiac contraction	Quail embryonic heart	Pulse laser (1875 nm)	Temporal (1–2 ms)	-	-	Jenkins et al. 2010**
Cardiac [Ca^2+^]_i_ increase	Rat neonatal cardiomyocytes	Pulse laser (1862 nm)	Temporal (3–4 ms)	-	Mitochondria	Dittami et al. 2011**
Ca^2+^-independent muscle contraction	Rat adult cardiomyocytes	CW laser (1455 nm)	Temporal (0.2–0.5 s)	Eu-TTA in a glass pipette	Sarcomere***	Oyama et al. 2012a
Ca^2+^-independent muscle contraction	Rat neonatal cardiomyocytes	CW laser (1455 nm)	Temporal (10 s)	Thermometer nanosheet	Sarcomere***	Shintani et al. 2015
Ca^2+^-independent muscle contraction	C2C12 myotube	AuNS (808 nm)	Temporal (0.5 s)	ER thermo yellow	Sarcomere***	Marino et al. 2017
Transporter speed-up	HeLa	CW laser (1455 nm)	Temporal (1 s)	Walking nanothermometer	Endosome/lysosome-transporting motors	Oyama et al. 2012b

This table summarizes responses of intact cells to temperature gradients introduced in this review. Responses of cells that were engineered to overexpress temperature-sensitive proteins are not contained

^*^These studies did not measure changes in temperature during optical stimulation, and the contribution of the temperature was unclear

^**^These studies did not measure changes in temperature during optical stimulation, but the contribution of the temperature was discussed

^***^The mechanism is considered to be the thermal activation of thin filament due to partial dissociation of Tm-Tn complex from actin filaments. Heating-enhanced Ca^2+^ binding to troponin C and/or myosin binding to actin filaments might be related (see text for the details)

Abbreviations: *[Ca*^*2*+^*]*_*i*_ intracellular [Ca^2+^], *CW* continuous wave, *ER* endoplasmic reticulum, *Eu-TTA* europium (III) thenoyltrifluoroacetonate trihydrate, *IP*_*3*_*Rs* inositol trisphosphate receptors, *SERCA* sarco-/endoplasmic reticulum Ca^2+^-ATPase, *TRP* transient receptor potential

Opto-thermal manipulation is effective to modulate multiple types of protein or process simultaneously, which is challenging in methods based on light-sensitive proteins and materials. On the other hand, for the same reason, selective targeting is not achieved when micrometer-scale heaters are used. To add selectivity, attaching nanoheaters to the targeted proteins is an effective strategy (Stanley et al. 2012; Iwaki et al. 2015).

Optical heating with NIR light is suitable for deep tissue applications. For instance, Miyako et al. injected carbon nanohorns with IR800CW under the thigh of frog expressing thermo TRP channels in nerves endogenously and successfully induced twitching of the paw via irradiation of 800-nm laser light from the outside of the frog body (Miyako et al. 2014). For deeper heating, radio-frequency magnetic field heating of nanoparticles has been adapted. Huang et al. used 6-nm manganese ferrite nanoparticles to activate temperature-sensing neurons in *C. elegans* for remote manipulation of the worm (Huang et al. 2010). Magnetothermal heating has also been demonstrated to excite neurons expressing TRPV1 in mouse brain (Chen et al. 2015).

## Perspectives

The discovery of thermo TRP channels led researchers to develop the thermal manipulation of neural activities, called “thermogenetics” (Bernstein et al. 2012; Ermakova et al. 2020). Other temperature-sensing systems that are uncovered yet could have the potential to be key components of the next method of advanced thermal manipulations and thermal therapies.

To understand the mechanism of temperature-sensing systems in cells, approaches using reconstituted systems composed of purified proteins are sometimes appropriate (e.g., see Ishii et al. 2019). One-by-one reconstitution of components while examining the thermal response of the system allows examination of the contribution of each component directly. Computational methods are also effective to explain the interactions of multiple temperature-sensitive proteins. Shintani et al. reproduced HSOs in numerical simulation of sarcomere by hypothesizing on multiple temperature effects on thick and thin filaments (Shintani et al. 2020).

The flow of media induced by convection needs to be considered with caution in microheating experiments (Tseeb et al. 2009), as the flow may stimulate cells mechanically. To examine the effect of convection, we apply a similar or stronger flow of media to the cells as a control and confirm that no obvious responses are induced in the cells as in the heating (Oyama et al. 2015a). Minimizing convection is an alternative approach by reducing the height of the imaging chamber down to ~ 10 μm, if allowed by the experimental design (Maeda et al. 2011). We also note that the temperature gradient causes a concentration gradient of biomolecules by the process known as thermophoresis or the Soret effect (Duhr and Braun 2006; Baaske et al. 2007; Budin et al. 2009; Fukuyama and Maeda 2020). Although thermophoresis has not been examined extensively in studies of cellular temperature-sensing, it is an attractive subject to reveal if and how thermophoresis is involved.

Cellular responses observed under the microscope may not represent those in tissues. On stiff basements such as glass, plastic, or other polymer-based dishes (> GPa), cellular morphology, gene and protein expression, and cellular functions such as migration differ from those on soft biomaterials that mimic soft tissues in vivo (1–1000 kPa) (van Helvert et al. 2018; Guimarães et al. 2020; Romani et al. 2021). Cells cultured on or within the soft biomaterials form unique multicellular 3D structures such as spheroids and organoids (Hofer and Lutolf 2021). Recently, Zhu et al. used star-shaped gold nanoparticles in MCF-7 tumor spheroid for heating and observed [Ca^2+^]_i_ increases in the targeted cells, followed by the propagation of Ca^2+^ waves to adjacent cells (Zhu et al. 2021).

Finally, there remains substantial room for the development of advanced optical heaters and thermometers. Intracellular thermometry reported 1 °C or greater local temperature gradients in both stimulated and non-stimulated cells at the organelle level, such as in nuclei (Okabe et al. 2012; Nakano et al. 2017) (see also Vu et al. 2021 for the controversial result), mitochondria (Okabe et al. 2012; Kiyonaka et al. 2013; Homma et al. 2015; Nakano et al. 2017; Huang et al. 2018, 2021; Chrétien et al. 2018, 2020; Savchuk et al. 2019; Di et al. 2021), and ER/SR (Kiyonaka et al. 2013; Arai et al. 2014; Itoh et al. 2016; Hou et al. 2017; Kriszt et al. 2017; Oyama et al. 2020). However, these experimental results largely contradict to the estimates based on the theories of macroscopic heat transfer, which expect the local temperature rises of the orders of 10^−4^ to 10^−5^ °C. For interested readers of this issue, we recommend Baffou et al.’s commentary (Baffou et al. 2014) and following communications (Kiyonaka et al. 2015; Suzuki et al. 2015; Baffou et al. 2015), a comprehensive discussion from both biological and physical viewpoints (Macherel et al. 2021), and our review article (Suzuki and Plakhotnik 2020). The issue is partially caused by the ambiguity in the physical parameters for intracellular heat diffusions. As demonstrated by the nanohybrids (Sotoma et al. 2021; Song et al. 2021), technological advances would provide further insights into the heterogeneous local heat transfer in more detail and how the heterogeneity is caused by, e.g., the intracellular architectures and their building blocks. Moreover, to reveal the physiological roles of intracellular temperature gradients (both spatial and temporal), methods of heating that can reproduce these local temperature gradients, or organelle-targeted nanoheaters, are desired. Simultaneous measurement of temperature and another parameter by a single probe will be a powerful approach to explore the heat-induced changes in the parameter. For instance, it was possible to measure the temperature sensitivity of the velocity of active transport directly in cells by the nanometry of individual luminescent nanothermometers (Oyama et al. 2012b). FNDs may be powerful probes for measuring multiple intracellular parameters such as electric and magnetic fields, pH, and protein dynamics in addition to the temperature (Fujisaku et al. 2019; Barry et al. 2020; Igarashi et al. 2020).

In this review, we introduced opto-thermal technologies and their applications for investigating cellular temperature-sensing. They have already been widely applied in the field of human, animal, and plant studies, and the number of studies is increasing. Ongoing advances of these technologies will reveal novel temperature-sensing systems and physiological significance of thermogenesis in cells and lead us to the development of advanced thermal therapies.
